# Case Report: Blood and cerebrospinal fluid mNGS-assisted diagnosis *Toxoplasma gondii* infection-associated with hemophagocytic syndrome and systemic lupus erythematosus

**DOI:** 10.3389/fmed.2025.1674391

**Published:** 2025-11-14

**Authors:** Xingyuan Chen, Xuegao Yu, Jiankai Deng, Juhua Yang, Peisong Chen

**Affiliations:** 1 Department of Laboratory Medicine, Guangxi Hospital Division of The First Affiliated Hospital, Sun Yat-sen University, Nanning, China; 2 Department of Laboratory Medicine, The First Affiliated Hospital, Sun Yat-sen University, Guangzhou, China; 3 Vision Medicals Co., Ltd., Guangzhou, China

**Keywords:** *Toxoplasma gondii* infection, hemophagocytic syndrome (HPS), systemic lupus erythematosus (SLE), metagenomic next-generation sequencing (mNGS), immunological disorder

## Abstract

**Background:**

Reactivation of latent *Toxoplasma gondii* (*T. gondii*) infection is more prevalent than primary infection in patients with autoimmune diseases. We present a rare case of systemic lupus erythematosus (SLE) and hemophagocytic syndrome (HPS) associated with *T. gondii* infection.

**Case presentation:**

We describe the case of a young girl with SLE and HPS who presented with fever, dyspnea, and pancytopenia. The patient's *T. gondii* infection was diagnosed through the detection of double-positive IgM and IgG antibodies. Metagenomic next-generation sequencing (mNGS) analysis of both plasma and cerebrospinal fluid (CSF) samples revealed a high concentration of *T. gondii* DNA. The patient demonstrated a positive response to a combined treatment regimen consisting of anti-Toxoplasma medications and glucocorticoids.

**Conclusions:**

Co-infection with uncommon pathogens is not uncommon in patients with autoimmune diseases. In individuals with immune disorders and positive *T. gondii* IgM antibodies, mNGS analysis of peripheral blood samples proves valuable in diagnosing disseminated *T. gondii* infection.

## Background

*Toxoplasma gondii* is a protozoan parasite that exhibits a complex life cycle and possesses a broad range of hosts, resulting in its worldwide distribution (1). Infection with *T. gondii* typically remains asymptomatic; however, it can cause congenital birth defects and encephalitis in individuals with compromised immune systems. Vertical transmission of tachyzoites from an infected mother to the fetus can occur during pregnancy, potentially leading to severe clinical consequences that vary depending on the timing of infection and the host species involved (2).

Reactivation of latent *T. gondii* infection primarily affects the central nervous system. Immunodeficient individuals are at risk of reviving latent infections and developing acute symptoms. The clinical presentation of cerebral toxoplasmosis is complex and lacks specificity. In pregnant women, the global prevalence of latent toxoplasmosis has been estimated at 33.8%, with the highest prevalence observed in South America (56.2%; 50.5%−62.8%) and the lowest in the Western Pacific region (11.8%; 8.1%−16.0%). Notably, the prevalence of latent toxoplasmosis is significantly higher in low-income and low Human Development Index (HDI) countries (3).

While clinicians generally possess a good understanding of toxoplasmosis in immunocompetent patients, identifying toxoplasmic encephalitis in immunocompromised patients remains challenging. Molecular assays, such as PCR-based methods, have been employed for pathogen detection; however, their utility is limited to specific pathogens. In contrast, metagenomic next-generation sequencing (mNGS) has the capability to directly identify thousands of pathogens from various samples, offering a promising approach for comprehensive pathogen detection.

## Case presentation

A 21 years old female presented with an acute exacerbation of a chronic course. She had been diagnosed with systemic lupus erythematosus (SLE) over 2 years ago and was transferred to our hospital due to septic shock, hemophagocytic syndrome, and acute left heart failure 1 month ago. Upon admission, the patient underwent relevant tests and examinations, which revealed SLE, acute left heart failure, pulmonary edema, combined infections, and acute renal failure requiring hemodialysis. The results of bone marrow aspiration indicated a high likelihood of hemophagocytic syndrome associated with infection.

The patient received multiple treatments including anti-infection therapy, pericardiocentesis and drainage, regular hemodialysis, transfusion of washed red blood cells, fresh frozen plasma, and fibrinogen to correct anemia and improve coagulation function. Additionally, she received transfusion of gammaglobulin and closed antibody to enhance the basic immune system, as well as albumin to correct hypoalbuminemia and multiple plasma cavities.

**Figure 1:**
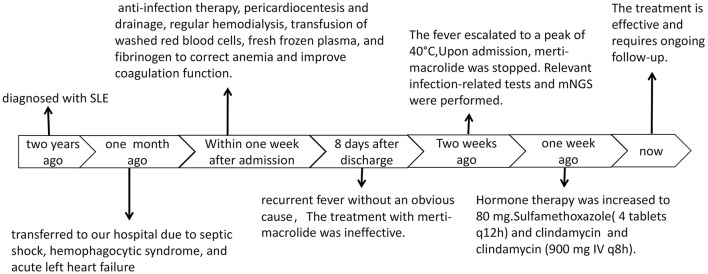
Clinical management flowchart for the patient.

Several weeks ago, the patient began experiencing recurrent fever without an obvious cause. Her temperature fluctuated between 37–38 °C, and she experienced episodes of physical hypothermia, with her temperature occasionally falling to normal levels. The fever escalated to a peak of 40 °C, prompting the patient to consult the outpatient clinic. Following the doctor's advice, the use of merti-macrolide was discontinued, but the fever persisted and was accompanied by a dry cough (Figure 1).

Tests for Influenza A and B viruses, Mycoplasma pneumoniae, fungal dextran (G test), Aspergillus, and Cryptococcus all yielded negative results. The EBV DNA and HCMV DNA tests showed results below the lower limit of detection. The Direct Coombs test was positive (2+), and the erythropoietin (EPO) level was 33.61 IU/L. Peripheral blood analysis revealed Heterozygous Lymphocyte Type I at 0.03% and oval Red Blood Cells (+++). The *Toxoplasma gondii* antibody test showed Toxo-IgM at 24.33 IU/mL and Toxo-IgG at >200.00 IU/mL. The Cytomegalovirus antibody test indicated CMV-IgM at 4.97 IU/mL and CMV-IgG at >250.00 IU/mL. The Rubella virus antibody test showed RVB-IgG at 14.80 IU/mL. Peripheral blood smears were negative for blood parasites.

Pathological examination of a bone marrow biopsy revealed generally normal bone marrow proliferation with lobulated nucleated megakaryocytes. There were a few cells with slightly irregular nuclei scattered among the hematopoietic tissues. Combined with the immunohistochemical results, the lesion was considered to be bone marrow lymphoid hyperplasia and macrophage activation. Hormone therapy was increased to 80 mg. An ultrasound examination showed an enlarged liver and spleen.

**Figure 2:**
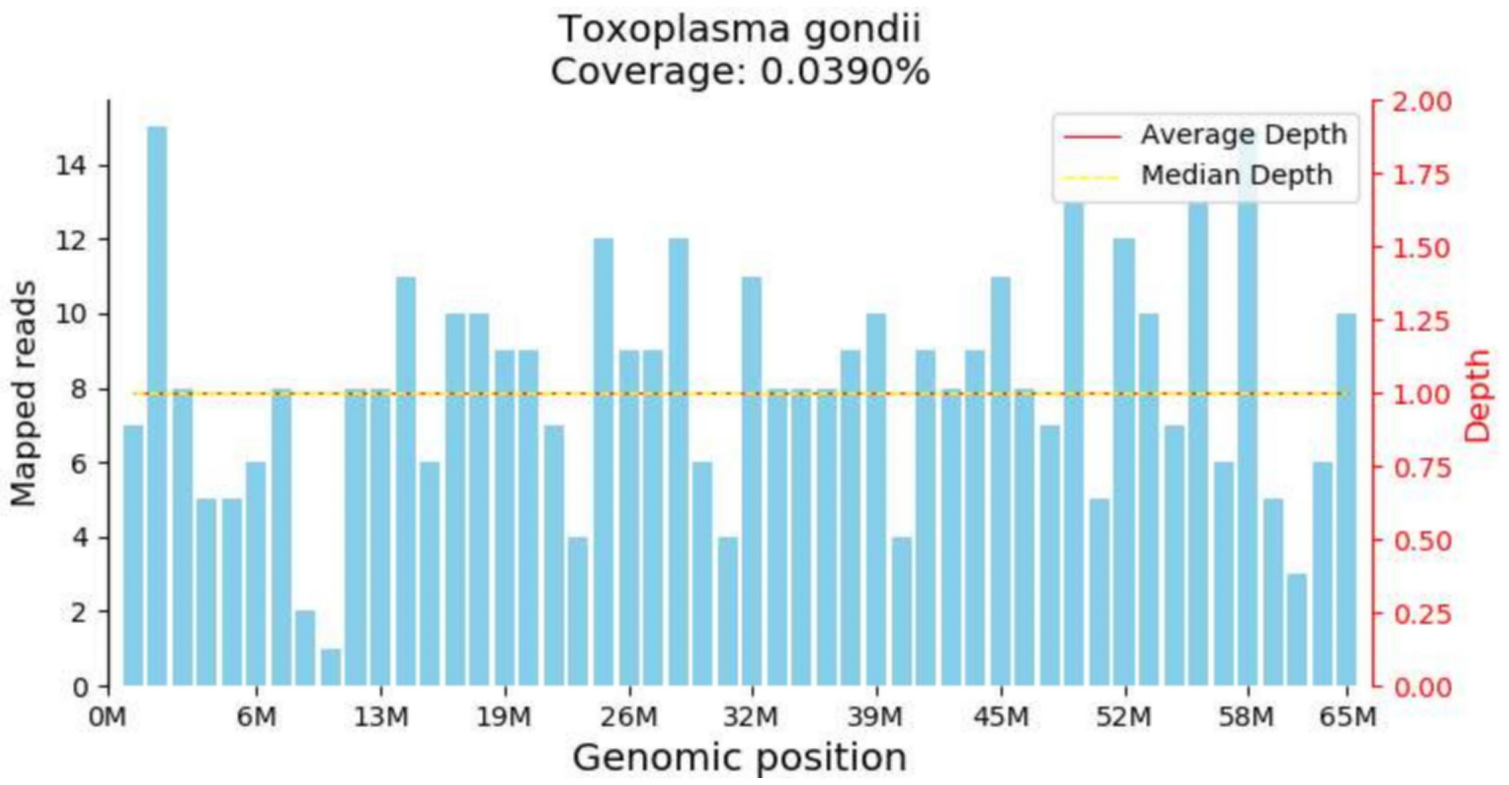
Metagenomic Nest-Generation Sequencing (mNGS) Detection of Toxoplasma gondii. The results showed extremely low genome coverage of 0.039%. Although this finding confirmed the presence of toxoplasma DNA in the sample, the extremely low coverage needs to be interpreted with caution.

**Figure 3:**
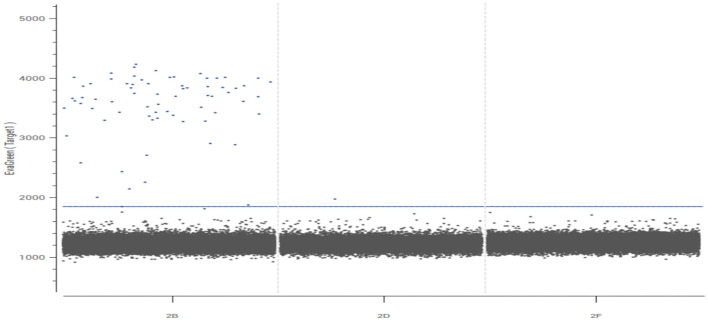
Validation results of Toxoplasma gondii by digital PCR. The dPCR plot displays the fluorescence amplitude of each individual partition (droplet or well). Two distinct clusters are evident: (1) a large cluster of partitions with low fluorescence intensity **(bottom left)**, corresponding to negative partitions that do not contain the target DNA; and (2) a clear cluster of partitions with high fluorescence intensity **(top right)**, identified as positive partitions that contain one or more copies of the target T. gondii DNA. The clear separation between these clusters confirms the specificity of the assay. The absolute quantification of the target DNA was calculated based on the proportion of positive partitions using the Poisson distribution.

Both peripheral blood and cerebrospinal fluid samples underwent pathogenic microorganism analysis using the Illumina NextSeq 550 high-throughput sequencing process. The mNGS analysis detected *Toxoplasma gondii* in both the peripheral blood and cerebrospinal fluid samples (Figure 2). The analysis yielded 448 sequence reads, with a coverage depth of 0.1% and a relative abundance of 99.7%. The taxonomic identification confidence was 99%.The sequencing run achieved a Q30 of 90%. The results were validated using Digital PCR with the primers LeftPrimer: GCCAGGACCCTTTGCTTTG and RightPrimer: TCATCTCCAGTCTTCGTTTCTCTAC (Figure 3). The patient was diagnosed with *Toxoplasma gondii* infection, which was successfully treated with oral clindamycin, resulting in a favorable therapeutic outcome. The patient responded well to Sulfamethoxazole (TMP 80 mg/SMX 400 mg, 4 tablets q12h) and clindamycin (900 mg IV q8h) against the infection and was able to discontinue immunosuppressive drugs.

## Discussion and conclusion

*Toxoplasma gondii* has a strong affinity for the brain and eyes. Studies have shown a correlation between *T. gondii* infection and increased risk of psychiatric disorders such as schizophrenia, bipolar disorder, and obsessive-compulsive disorder (4, 5). While the immune response triggered by infection helps control the parasite, it can also have detrimental effects on neuronal function. Interferon-gamma (IFNγ) can disrupt neurogenesis, inhibit microglia function, lead to neurodegeneration, and interfere with dendritic processes through upregulation of major histocompatibility complex (MHC). These neurological changes can also manifest as behavioral differences (6). In the case of this patient, *Toxoplasma gondii* was detected in the cerebrospinal fluid, but fortunately, no neurological symptoms were observed.

Hemophagocytic syndrome (HPS) is a group of syndromes characterized by abnormal proliferation of tissue cells and the phagocytosis of various blood cells, excluding multinucleated giant histiocytes. Common triggers of HPS include infections, tumors, and autoimmune diseases. The disease often presents with persistent fever, hepatosplenomegaly, and other symptoms, which can be improved with medication. However, if not promptly treated, HPS can be fatal.

Systemic lupus erythematosus (SLE) is an autoimmune disease that affects multiple systems and organs and is characterized by the presence of multiple autoantibodies. *Toxoplasma gondii* has been identified as a risk factor for the prevalence and severity of SLE (7). In patients with both HPS and SLE, with positive *T. gondii* IgM antibodies, the use of metagenomic next-generation sequencing (mNGS) analysis of a peripheral blood sample can be helpful in diagnosing disseminated *T. gondii* infection (8). The dynamic changes detected through serological testing for *T. gondii* IgM and IgG antibodies further support the inference that the patient experienced a primary *T. gondii* infection. In this case, the patient had both an infectious and immunological disease, which could have acted as triggers or exacerbated the symptoms of HPS.

Diagnosis of toxoplasmosis primarily relies on laboratory tests. The most direct evidence is the detection of tachyzoites in body fluids or tissues using microscopic techniques. However, this method is time-consuming and requires skilled personnel to obtain reliable results. Serologic tests for *T. gondii* antibodies IgM and IgG are commonly used, but they may not accurately diagnose immunocompromised transplant patients based on serologic findings alone. Continuous quantitative PCR assays can detect changes in the *Toxoplasma gondii* load and help document the response to treatment.

Metagenomic next-generation sequencing (mNGS) is a revolutionary method for detecting DNA and RNA, enabling rapid and accurate identification of pathogenic organisms in a sample. This characteristic gives it significant potential in diagnosing challenging, critical, and rare infections. Immunocompromised patients are at an increased risk of infection by rare pathogens and often undergo mNGS testing in clinical settings (9). Several studies have shown the potential of microRNAs as molecular markers for the development of diagnostic tools for human toxoplasmosis (10). In this case, the combination of HPS and SLE may have overshadowed the possibility of rare pathogen infections. mNGS maximizes the ability to detect rare pathogen infections in complex cases. However, mNGS also has limitations, such as high costs, demanding expertise requirements, and accessibility issues. Therefore, it is essential to establish clear clinical application pathways and foster multidisciplinary collaboration to develop standardized operational procedures, reporting interpretation criteria, and result verification systems, ultimately maximizing its value in enhancing public health.

*Toxoplasma gondii* is an opportunistic parasitic protozoan that can cause severe toxoplasmic encephalitis when the host's immune system is compromised (11). In this particular case, the patient's cerebrospinal fluid showed low sequence counts of *Toxoplasma gondii* DNA, but no obvious symptoms of brain infection were observed. This may be attributed to low pathogen loads in the brain, highlighting the importance of early detection of toxoplasmosis infection. Due to the prolonged and complicated diagnostic process, the patient presented with mild anxiety and depression symptoms. The exact route of Toxoplasma infection remains undetermined. As a young woman, the patient expressed significant concern regarding the potential impact of the infection on her future fertility. Healthcare providers proactively offered easy-to-understand explanations about the disease and standardized reproductive health guidance. The patient actively cooperated, strictly adhered to the medication regimen, and proactively adjusted her lifestyle.

The long-term impact of *Toxoplasma gondii* infection on patients with autoimmune diseases is a multidimensional and dynamic process, primarily stemming from the complex and persistent interaction between the parasite and the host's immune system. Toxoplasma infection may exacerbate autoimmune dysregulation through mechanisms such as “molecular mimicry” and “bystander activation.” Additionally, the potential risk of drug interactions constitutes another long-term concern, while the immunosuppressed state increases the risk of reactivation of latent toxoplasmosis. For patients with autoimmune diseases concurrent with Toxoplasma infection, long-term, interdisciplinary follow-up and monitoring are recommended.

Considering the relatively high prevalence of *Toxoplasma gondii* in the Chinese population, it should be a concern for patients with autoimmune diseases (12). Testing for *Toxoplasma gondii* DNA is recommended when IgM anti-*T. gondii* antibodies are positive. Patients with autoimmune diseases are susceptible to rare pathogen infections, and mNGS can play a crucial role in achieving prompt and accurate etiological diagnoses. This, in turn, can facilitate optimized antimicrobial therapy, reduce healthcare costs, improve the doctor-patient relationship, and mitigate drug-related toxicities. This case indirectly demonstrates the value of applying the sensitivity and specificity of mNGS in clinical practice. Additional studies are necessary to verify these results in larger cohorts.

## Data Availability

The datasets presented in this article are not readily available due to patient confidentiality. Requests to access the datasets should be directed to 1411263743@qq.com.
